# Myoblast-conditioned media improve regeneration and revascularization of ischemic muscles in diabetic mice

**DOI:** 10.1186/s13287-015-0063-8

**Published:** 2015-04-12

**Authors:** Magdalena Kozakowska, Jerzy Kotlinowski, Anna Grochot-Przeczek, Maciej Ciesla, Bartosz Pilecki, Rafal Derlacz, Jozef Dulak, Alicja Jozkowicz

**Affiliations:** Department of Medical Biotechnology, Faculty of Biochemistry, Biophysics and Biotechnology, Jagiellonian University, Gronostajowa 7, Krakow, 30-387 Poland; R&D Department, Adamed Ltd, Pienkow 149, Czosnow, 05-152 Poland; Department of Metabolic Regulation, Institute of Biochemistry, Faculty of Biology, University of Warsaw, Miecznikowa 1, Warsaw, 02-096 Poland; Malopolska Centre of Biotechnology, Jagiellonian University, Krakow, Poland

## Abstract

**Introduction:**

Diabetes is associated with reduced expression of heme oxygenase-1 (HO-1), a heme-degrading enzyme with cytoprotective and proangiogenic properties. In myoblasts and muscle satellite cells HO-1 improves survival, proliferation and production of proangiogenic growth factors. Induction of HO-1 in injured tissues facilitates neovascularization, the process impaired in diabetes. We aimed to examine whether conditioned media from the HO-1 overexpressing myoblast cell line can improve a blood-flow recovery in ischemic muscles of diabetic mice.

**Methods:**

Analysis of myogenic markers was performed at the mRNA level in primary muscle satellite cells, isolated by a pre-plate technique from diabetic db/db and normoglycemic wild-type mice, and then cultured under growth or differentiation conditions. Hind limb ischemia was performed by femoral artery ligation in db/db mice and blood recovery was monitored by laser Doppler measurements. Mice were treated with a single intramuscular injection of conditioned media harvested from wild-type C2C12 myoblast cell line, C2C12 cells stably transduced with HO-1 cDNA, or with unconditioned media.

**Results:**

Expression of HO-1 was lower in muscle satellite cells isolated from muscles of diabetic db/db mice when compared to their wild-type counterparts, what was accompanied by increased levels of Myf5 or CXCR4, and decreased Mef2 or Pax7. Such cells also displayed diminished differentiation potential when cultured *in vitro*, as shown by less effective formation of myotubes and reduced expression of myogenic markers (myogenic differentiation antigen - myoD, myogenin and myosin). Blood flow recovery after induction of severe hind limb ischemia was delayed in db/db mice compared to that in normoglycemic individuals. To improve muscle regeneration after ischemia, conditioned media collected from differentiating C2C12 cells (control and HO-1 overexpressing) were injected into hind limbs of diabetic mice. Analysis of blood flow revealed that media from HO-1 overexpressing cells accelerated blood-flow recovery, while immunohistochemical staining assessment of vessel density in injected muscle confirmed increased angiogenesis. The effect might be mediated by stromal-cell derived factor-1α proangiogenic factor, as its secretion is elevated in HO-1 overexpressing cells.

**Conclusions:**

In conclusion, paracrine stimulation of angiogenesis in ischemic skeletal muscle using conditioned media may be a safe approach exploiting protective and proangiogenic properties of HO-1 in diabetes.

## Introduction

Heme oxygenase-1 (HO-1) is an enzyme degrading heme to three products: carbon monoxide (CO), ferrous ions (Fe^2+^) and biliverdin, the latter subsequently converted to bilirubin by biliverdin reductase. Enzymatic activity of HO-1 attenuates oxidative stress and inflammatory reaction, augments angiogenesis, increases cell survival, and influences cell cycle [1]. The cytoprotective and anti-apoptotic effects of HO-1 have been demonstrated in different cell types exposed to reactive oxygen species, proinflammatory cytokines, heat shock, or serum deprivation [1]. Furthermore, HO-1 upregulates expression of vascular endothelial growth factor (VEGF), the major proangiogenic mediator [2], and is necessary for proper function of stromal cell-derived factor-1α (SDF-1α), the cytokine responsible for homing of proangiogenic progenitors and activation of mature endothelial cells [3]. Beneficial effects of HO-1 have also been shown in cutaneous wound healing, tissues subjected to ischemia-reperfusion injury and in transplanted organs [4-6].

Proangiogenic properties of HO-1 were shown to improve the restoration of blood flow in ischemic skeletal muscles and facilitate muscle regeneration [6]. Indeed, the role of HO-1 in muscle tissue is not restricted to stimulation of neovascularization in response to ischemia. HO-1 also influences functioning of progenitor cells - muscle satellite cells (mSCs) and myoblasts [7] - which are responsible for formation of new myofibers after muscle degeneration or injury [8,9]. We have shown that activation of HO-1 increases myoblast proliferation and improves their viability in oxidative stress [7]. On the other hand, constitutive activity of HO-1 decreases myoblast differentiation via CO-dependent inhibition of CCAAT/enhancer-binding protein-δ followed by reduced myogenic differentiation antigen (myoD) transcription, as well as through upregulation of SDF-1α and downregulation of myomirs, the muscle-specific microRNAs [7]. Indeed, after injection of murine myoblasts (C2C12 cell line) overexpressing HO-1 into gastrocnemius muscle of immunocompromized mice, the viability and proliferation of the transplanted cells were improved but their differentiation and formation of myofibers were blocked. Instead, HO-1 overexpressing myoblasts formed hyperplastic tumors infiltrating the surrounding tissues [7]. Hence, although HO-1 acts as a cytoprotective and proangiogenic protein which could be targeted to facilitate vascularization of ischemic muscle, especially in diabetic patients characterized by oxidative stress and impaired angiogenesis, there is a need to elaborate a therapeutic protocol that can minimize the risk of adverse effects.

Diabetes is a chronic disease leading to endothelial dysfunction along with impairment of the microcirculation [10] and inhibition of blood flow restoration after injury [11-15]. Reduced angiogenesis also affects regeneration of skeletal muscle in diabetic individuals [11], the process additionally impaired by dysfunction of muscle progenitor cells [11,16-18]. Altogether, this may cause the development of non-healing diabetic wounds also comprising muscle degeneration, known as diabetic ulcer syndrome, which eventually may lead to the necessity for toe or leg amputation [19]. Enhanced expression of HO-1 [4,20] or SDF-1α [12,19,21] can overcome at least some deleterious diabetic complications. However, it has not been determined so far whether the paracrine activity of myoblasts overexpressing HO-1 may improve neovascularization of ischemic muscles in diabetes.

In the present study, we investigated the effect of conditioned media harvested from HO-1 overexpressing C2C12 myoblast cell line on blood flow recovery and muscle regeneration in diabetic mice subjected to hind limb ischemia (HLI).

## Methods

### Cell culture

Primary mSCs were isolated from normoglycemic wild-type or diabetic db/db mice of C57BLKS background (Taconic, Denmark, Ry), according to a pre-plate technique [7]. Experiments were performed on pre-plate number 6, which represented a population of muscle-derived cells enriched in satellite cells. For a routine culture the Dulbecco’s modified Eagle’s medium supplemented with glucose 25 mmol/L (PAA Laboratories, Austria), supplemented with 10% fetal calf serum (FCS; PAA Laboratories), 10% horse serum (PAA Laboratories), penicillin (100 U/mL; Sigma-Aldrich, USA), streptomycin (100 μg/mL; Sigma-Aldrich) and 0.05% chicken embryo extract (Accurate Chemicals, USA) was used. When cells reached 90 to 100% confluence, differentiation was induced by a 4-day culture in differentiation medium (DM) - routine culture medium containing 2% horse serum, instead of 10% fetal calf serum and 10% horse serum. As a control, growth medium (GM) was used, with 20% fetal calf serum.

Modified murine myoblast cells expressing control reporter genes coding for luciferase and green fluorescent protein (C2C12-Luc-GFP) or additionally overexpressing HO-1 (C2C12-Luc-GFP-HO1), were generated from C2C12 myoblast cell line by stable retroviral transduction [7]. Cells were cultured and differentiated as described previously [7]. Briefly, for a routine culture and as a control GM, the Dulbecco’s modified Eagle’s medium supplemented with glucose (25 mmol/L), 10% fetal calf serum, penicillin (100 U/mL), and streptomycin (100 μg/mL) was used. DM contained 2% horse serum instead of 10% fetal calf serum.

To prepare conditioned media from C2C12-Luc-GFP and C2C12-Luc-GFP-HO1 cells, the GM or DM media were changed on EBM-2 (Lonza, Switzerland) with 0.5% fetal calf serum, penicillin (100 U/mL) and streptomycin (100 μg/mL) after the first or fourth day of differentiation, and cells were cultured further for 24 hours.

### Hind limb ischemia in mice

All procedures were performed in accordance with national and European legislations, after approval by the Local Ethical Committee for Animal Experimentation in Krakow. Animals were kept under controlled environmental conditions (12-hour light/dark cycle at ~23°C), with water and food available *ad libitum*.

To induce HLI, a double ligation of the superficial left femoral artery and vein was performed in 18 male db/db mice. Next day, animals were randomly divided into three groups for treatment with different types of conditioned media (six individuals per group; media in a total volume of 50 μL injected intramuscularly into three sites of ischemic gastrocnemius muscle): i) unconditioned media (fresh EBM-2 + 0.5% FCS); ii) conditioned media (EBM-2 + 0.5% FCS) from differentiated C2C12-Luc-GFP cells, harvested on the fifth day of differentiation; iii) conditioned media (EBM-2 + 0.5% FCS) from C2C12-Luc-GFP-HO1 cells, harvested on the fifth day of differentiation.

The superficial blood flow of the ischemic and contralateral leg was analyzed using laser Doppler Blood Perfusion Imager PeriScan PIM2 (Perimed, Sweden). The ratio between blood flow in the ischemic leg and the contralateral leg was calculated and used as an index of blood flow recovery. Measurements were performed before HLI (−1 day of experiment), on the day of media injection (day 0) and on days 7, 14 and 28 after HLI. On the last day of experiment animals were euthanized, and peripheral blood, bone marrow and gastrocnemius muscle were collected for further analyses.

### Blood morphology and creatine phosphokinase activity

Blood for analysis of morphology was collected from the tail vein and analyzed with ABC Vet (Horiba ABX, Japan). Serum was collected for analysis of creatine phosphokinase activity, performed with the EnzyChrom Creatine Kinase Assay Kit (BioAssays Systems, USA), according to the manufacturer’s instruction.

### Analysis of bone marrow-derived proangiogenic progenitor cells

Peripheral blood and bone marrow were collected and centrifuged (400 *g*, 10 minutes), and erythrocytes were lysed with Pharm Lyse (BD Bioscience, Singapore). The cell pellet was suspended in RPMI medium (PAA Laboratories) with 2% FCS and stained with anti-Sca-1-FITC, anti-KDR-APC, and anti-CD45-Cy7 antibodies (all diluted 1:200; BD Bioscience) or with appropriate izotype controls for 30 minutes. Afterwards, non-conjugated antibodies were washed out, and cells were fixed with 1% paraformaldehyde (POCh, Poland). CD45^−^Sca-1^+^KDR^+^ cells were analyzed using the LSRII flow cytometer with FACS Diva software (BD Bioscience).

### Histological analysis and immunohistochemical staining

Gastrocnemius muscles were fixed in OCT freezing medium (Leica Microsystems Germany). Sections (8 μm) were stained with hematoxylin for 15 minutes (Sigma Aldrich), rinsed with tap water for 15 minutes, counterstained with eosin (Sigma Aldrich) for 15 seconds, dehydrated with Ottix Shaper (Diapath, Italy) for 1 minute, Ottix Plus (Diapath) for 10 minutes, and mounted in Canadian balsam (Paul Marienfeld GmbH, Germany). Regenerating myofibers were analyzed in 10 different fields of view (magnification 200×) per section.

Frozen sections for CD31 (capillaries) and α-smooth muscle actin (αSMA; arterioles) analysis were fixed with acetone (POCh) or 2% paraformaldeyde (POCh), respectively, and blocked with 10% goat serum (Sigma Aldrich), 0.05% Tween (Sigma Aldrich), and 0.1% Triton X-100 (Sigma Aldrich) in phosphate-buffered saline. Sections were incubated for 1.5 hours with anti-CD31 antibody (BD Bioscience) diluted to the final concentration of 156.25 ng/mL or with anti-αSMA antibody (Abcam, UK) diluted 1:200. Afterwards secondary antibodies conjugated with rhodamine (1:1000; Cappel, USA) or Alexa488 (1:400) were applied for 45 minutes for CD31 and αSMA staining, respectively. The number of capillaries were analyzed in 10 different fields of view (magnification 200×) per section, whereas the number of arterioles were analyzed per mm^2^ of a section, with ImageJ program (National Institutes of Health, USA).

### Gene expression analysis

Isolation of RNA from gastrocnemius muscle, bone marrow, and satellite cells followed by reverse transcription and quantitative PCR were performed as previously described [7]. Primers used in quantitative PCR reactions are presented in Table 1.

**Table 1:** **Sequence of primers used in the research**

**Gene**	**Sequence of starters**	
EF2	forward	5′ - GAC ATC ACC AAG GGT GTG CAG - 3′
	reverse	5′ - TCA GCA CAC TGG CAT AGA GGC - 3′
HO-1	forward	5′ - GTG GAG MCG CTT YAC RTA GYG C - 3′
	reverse	5′ - CTT TCA GAA GGG YCA GGT GWC C - 3′
SDF-1α	forward	5′ - AAT TTC GGG TCA ATG CAC AC - 3′
	reverse	5′ - GTG ACG GTA AGC CAG TCA GC - 3′
CXCR4	forward	5′ - AAA CCT CTG AGG CGT TTG GT - 3′
	reverse	5′ - AGC AGG GTT CCT TGT TGG AG - 3′
CXCR7	forward	5′ - CTG AGG TCA CTT GGT CGC TC - 3′
	reverse	5′ - TGC ACA GTG TCC ACC ACA AT - 3′
VEGF	forward	5′ - ATG CGG ATC AAA CCT CAC CAA GGC - 3′
	reverse	5′ - TTA ACT CAA GCT GCC TCG CCT TGC - 3′
VEGF-R1	forward	5′ - GCA CCT ATG CST GCA GAG C - 3′
	reverse	5′ - TCT TTC AAT AAA CAG CGT GCT G - 3′
VEGF-R2	forward	5′ - CCT CAC CTG TTT CCT GTA TGG AG - 3′
	reverse	5′ - GAK GCC ACA GAC TCC CTG C - 3′
Myf5	forward	5′ - CCT GTC TGG TCC CGA AAG AAC - 3′
	reverse	5′ - GAC GTG ATC CGA TCC ACA ATG - 3′
MEF2	forward	5′ - CAG GCG CTA TGG GTC ATC TG - 3′
	reverse	5′ - GCT ACT TGG ATT GCT GAA CTG C - 3′
myoD	forward	5′ - GCT GCC TTC TAC GCA CCT G - 3′
	reverse	5′ - GCC GCT GTA ATC CAT CAT GC - 3′
myogenin	forward	5′ - CAG TAC ATT GAG CGC CTA CAG - 3′
	reverse	5′ - GGA CCG AAC TCC AGT GCA T - 3′
myosin	forward	5′ - CAC TTT GGC ACT ACG GGG AAA C - 3′
	reverse	5′ - GCC ATC AGC TCT TCC TGG TCA T - 3′
Pax7	forward	5′ - CAA CCA CAT GAA CCC TGT CA - 3′
	reverse	5′ - GAG ATG GAG GAA GCC GAG TC - 3′

HO-1, heme oxygenase-1; myoD, myogenic differentiation antigen; SDF-1α, stromal cell-derived factor-1α; VEGF, vascular endothelial growth factor.

### Enzyme-linked immunosorbent assay

Concentrations of SDF-1α and VEGF in conditioned media from C2C12-Luc-GFP and C2C12-Luc-GFP-HO1 cells were measured using an enzyme-linked immunosorbent assay kit (R&D Systems, USA) according to the manufacturer’s instructions.

### Mass spectroscopy

Conditioned media from C2C12-Luc-GFP and C2C12-Luc-GFP-HO1 cells were lyophilized and mass spectroscopy was performed in triplicate with Synapt 2 HDMS PLGS 2.4 (Waters, USA) and protein database UniProt. The sample (800 ng) was administered on a column with internal protein standard of yeast alcohol dehydrogenase (50 to 75 fmol). Changes between samples were analyzed with Waters Expression E application.

### Statistical analysis

Results are expressed as mean ± standard error of the mean. Two tailed Student’s *t* test was used for comparison of two groups, while one-way analysis of variance with Bonferroni post-test was applied for comparison of multiple groups.

## Results

### Disrupted expression of myogenic markers in muscle satellite cells isolated from diabetic mice

**Figure 1:**
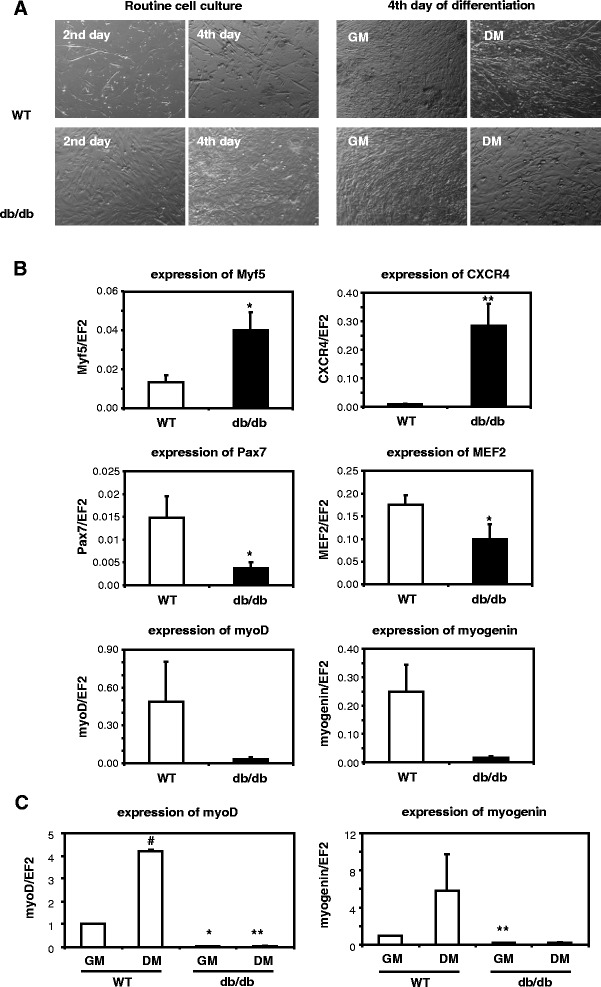
Muscle satellite cells (mSCs) isolated from wild-type (WT) or diabetic (db/db) mice and cultured in routine culture conditions (second and fourth day of cell culture, growth medium (GM)) and during differentiation (fourth day of differentiation, differentiation medium (DM)). **(A)** Morphology of mSCs cultured for 2 or 4 days in GM or for 4 days in DM; representative photos, magnification 100×. **(B)** Expression of Myf5, CXCR4, Pax7, Mef2, myoD and myogenin mRNAs in subconfluent mSCs isolated from WT and db/db mice and cultured in GM; quantitative RT-PCR (n = 5 to 13). **(C)** Expression of myoD and myogenin mRNAs in mSCs cultured for 5 days in GM or DM; quantitative RT-PCR (n = 2). EF2 served as an internal control. Each bar represents mean + SEM. *P < 0.05, **P < 0.01, versus WT; ^#^ *P* < 0.05, versus cells cultured in GM. myoD, myogenic differentiation antigen.

Primary mSCs isolated from wild-type and db/db mice showed the same morphology in a routine *in vitro* culture, but different growth characteristics; although db/db cells reached confluence a few days earlier than their wild-type counterparts, they formed elongated tubes less often in a routine cell culture (Figure 1A). Accordingly, the quantitative RT-PCR analysis of gene expression revealed significantly increased levels of Myf5 transcription factor (an early marker of activated, proliferating myoblasts) and CXCR4 receptor (associated with enhanced migration of myoblasts and a receptor for the myogenic mitogen SDF-1α) in cultured primary cells isolated from db/db mice (Figure 1B). Additionally, in diabetic mSCs, reduced expression of Pax7 (the transcription factor present in quiescent and proliferating but not in differentiating muscle progenitors) was observed (Figure 1B). Also, markers characteristic for differentiating myoblasts, namely Mef2, myoD and myogenin, were downregulated in cells derived from diabetic mice in comparison to mSCs isolated from wild-type animals (Figure 1B).

It appears, that lower expression of these myogenic differentiation factors may reflect a reduced capacity of mSCs to mature into myotubes during *in vitro* induced differentiation (Figure 1A). The morphological assessments were supported by analysis of differentiation markers. When cells were cultured for 4 days in conditions that promote differentiation, expression of myoD and myogenin were upregulated in the wild-type cells, but not in the cells isolated from db/db individuals (Figure 1C). Taken together, analysis of gene expression suggests that in diabetic mice the activation and proliferation of mSCs are efficient, but differentiation and formation of myotubes can be disturbed.

### Decreased expression of heme oxygenase-1 in muscle satellite cells isolated from db/db mice

**Figure 2:**
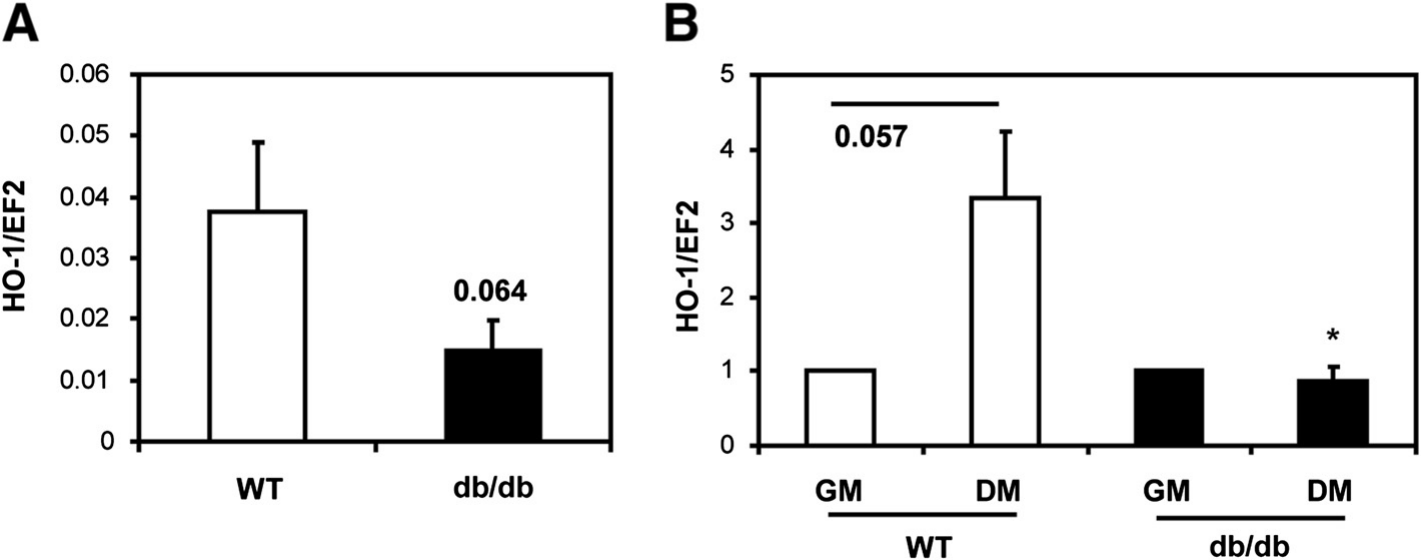
Expression of heme oxygenase-1 (HO-1) mRNA in muscle satellite cells (mSCs) isolated from wild-type (WT) and diabetic (db/db) mice. **(A)** Subconfluent cells cultured in growth medium (GM). **(B)** Cells cultured for 5 days in GM or differentiation medium (DM): expression of HO-1 in differentiated cells (DM) is shown as a proportion of that in undifferentiated cells (GM); quantitative RT-PCR (n = 11 to 13). EF2 served as an internal control. Each bar represents mean + SEM. *P < 0.05, versus WT.

Regeneration of ischemic muscles depends on differentiation of myoblasts, as well as on formation of new blood vessels [22]. Both myogenesis and angiogenesis are known to be regulated by HO-1 [1,7]. We examined the expression of HO-1 at the mRNA level either in subconfluent primary mSCs or in the cells subjected to differentiation. In a routine cell culture the HO-1 level showed a tendency to be decreased in mSCs isolated from db/db mice in comparison to that from wild-type individuals (*P* = 0.064; Figure 2A). Under differentiating conditions, expression of HO-1 was upregulated in wild-type cells (*P* = 0.057), whereas no such upregulation was observed in db/db mSCs (Figure 2B).

### Effect of heme oxygenase-1 overexpression on production of proangiogenic factors

In the next experiments we investigated whether forced expression of HO-1 upregulates the production of proangiogenic growth factors in myoblasts. To obtain high and reproducible expression of HO-1 we transduced the murine myoblast cell line (C2C12 cells) with HO-1 transgene using retroviral vectors. Previously we have demonstrated that such stable overexpression of HO-1 exerted a potent effect on differentiation, viability and proliferation of these cells [7]. Here we have examined whether the constitutively increased level of HO-1 can also influence the paracrine proangiogenic properties of C2C12 myoblasts.

**Figure 3:**
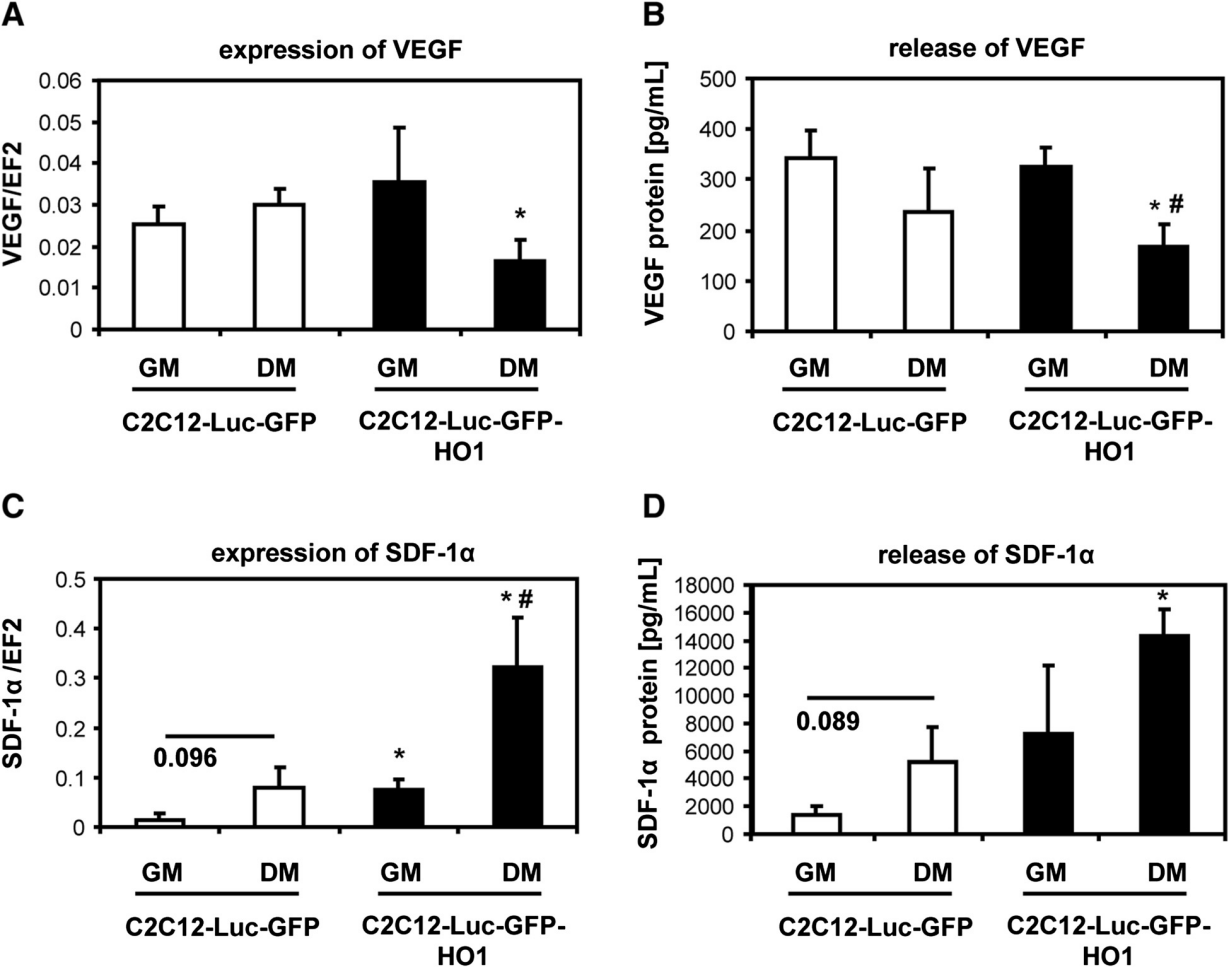
Production of vascular endothelial growth factor (VEGF) and stromal cell-derived factor-1α (SDF-1α) by C2C12-Luc-GFP and C2C12-Luc-GFP-HO1 cells cultured in growth media (GM) or differentiation media (DM). **(A)** Expression of VEGF mRNA; quantitative RT-PCR (n = 3). **(B)** Secretion of VEGF protein to conditioned media; enzyme-linked immunosorbent assay (ELISA) (n = 5). **(C)** Expression of SDF-1α mRNA; quantitative RT-PCR (n = 3). **(D)** Secretion of SDF-1α protein to conditioned media; ELISA (n = 5). EF2 served as an internal control. Each bar represents mean + SEM; ^*^ *p* < 0.05 vs. C2C12-Luc-GFP cells. ^#^ *P* < 0.05, versus cells cultured in GM.

We have focused on two major proangiogenic factors - VEGF and SDF-1α. Under routine culture conditions in GM the expression of VEGF was similar in C2C12-Luc-GFP and C2C12-Luc-GFP-HO1 cells. Likewise, induction of myoblast maturation in DM did not affect VEGF in control cells. Expression of VEGF mRNA was, however, decreased in differentiated myoblasts overexpressing HO-1 (Figure 3A). Release of VEGF protein to the culture medium was similarly decreased in such cells (Figure 3B). Concomitantly, HO-1 overexpression significantly increased production of SDF-1α, the effect especially pronounced in differentiated cells, both at mRNA (Figure 3C) and protein (Figure 3D) levels. Thus, media harvested from differentiated C2C12-Luc-GFP-HO1 cells contained reduced concentrations of VEGF (two-fold) but much higher concentrations of SDF-1α (15-fold) than that harvested from control undifferentiated C2C12-Luc-GFP cells.

To analyze secretome of C2C12 cells more comprehensively we performed a mass spectroscopy analysis of conditioned media harvested from differentiated C2C12-Luc-GFP and C2C12-Luc-GFP-HO1 cells. The results showed that overexpression of HO-1 led to complete blockage of the release of pigment epithelium-derived factor (PEDF), a potent inhibitor of angiogenesis (Table 2). Similar inhibition was found for biglycan (an extracellular matrix protein which can bind VEGF, increasing its storage or stabilization in the matrix and thereby regulating the VEGF signaling) and for follistatin-related protein-1 (a protein which is supposed to play a promyogenic and proangiogenic role) (Table 2). Conditioned media harvested from C2C12-Luc-GFP-HO1 cells also contained a reduced concentration of fibronectin (Table 2). On the other hand, overexpression of HO-1 resulted in induction of protein disulfide isomerase (a protein known to stimulate neovascularization of ischemic tissues), and in significant upregulation of macrophage migration inhibitory factor, peptidyl-prolyl cis-trans isomerase A, haptoglobin, and galectin-1, which are associated with enhanced angiogenesis (Table 2). Hence, mass spectroscopy analysis suggests that overexpression of HO-1 influences a composition of conditioned media, regulating release of proteins related to neovascularization with a possible shift of balance toward proangiogenic factors. Some HO-1 regulated proteins are also known to influence myogenesis (Table 2).

**Table 2:** **Proteins which concentrations differ significantly when harvested from C2C12-Luc-GFP and C2C12-Luc-GFP-HO1 cells**

	**Protein**	**Fold of change HO-1:GFP**	**Properties of protein**				**Selected references**
			**Angiogenesis**		**Myogenic**		
			**Pro**	**Anti**	**Pro**	**Anti**	
Downregulated in C2C12-Luc-GFP-HO1 cells	PEDF	only in GFP		+			[57,62]
	BGN	only in GFP		+	+		[67-69]
	FRP1	only in GFP	+		+		[66,70]
	Fibronectin	0.2	+		+		[65,71]
Upregulated in C2C12-Luc-GFP-HO1 cells	PDI	only in HO-1	+				[72]
	PPIase A	2.59	+			+	[58,73]
	MIF	3.06	+			+	[59,63]
	Haptoglobin	1.72	+				[60]
	Galectin-1	1.86	+		+		[61,64,74]

Mass spectroscopy analysis. Major activities related to angiogenesis and myogenesis are indicated, based on selected references. BGN, biglycan; FRP1, follistatin-related protein 1; GFP, green fluorescent protein; HO-1, heme oxygenase-1; MIF, macrophage migration inhibitory factor; PDI, protein disulfide isomerase; PEDF, pigment epithelium derived factor; PPIase A, peptidyl-prolyl cis-trans isomerase A.

### Effect of conditioned media from heme oxygenase-1 overexpressing C2C12 cells on recovery after hind limb ischemia

**Figure 4:**
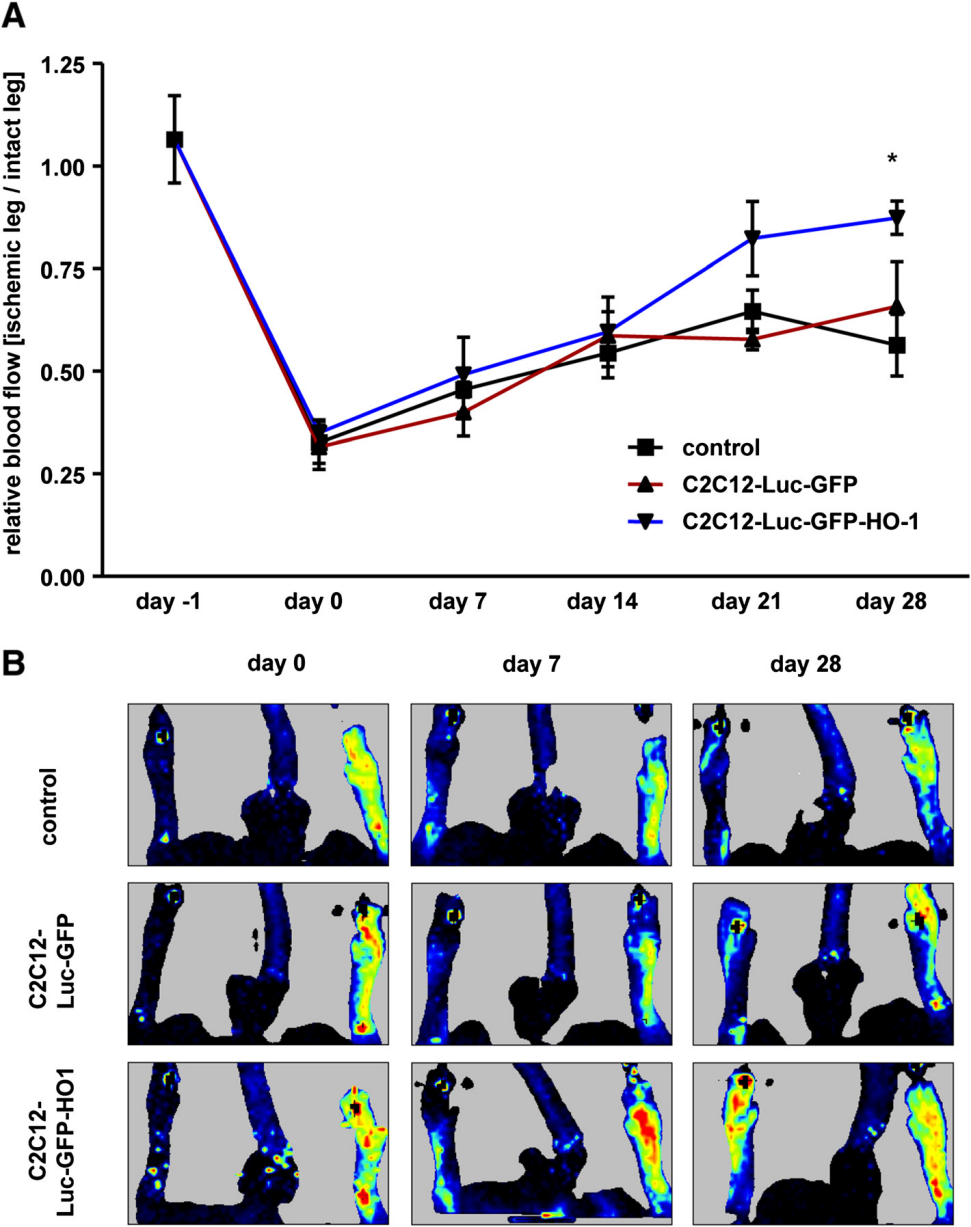
Regeneration of blood flow after hind limb ischemia in db/db mice treated with conditioned media from C2C12-Luc-GFP, C2C12-Luc-GFP-HO1 cells or nonconditioned control media. **(A)** Quantitative analysis of blood flow (n = 5 to 6). Each point represents mean ± SEM; *P < 0.05, versus control. **(B)** Representative pictures showing blood flow analysis (dark blue = low blood flow, red = high blood flow). Laser Doppler measurements.

Overexpression of HO-1 in C2C12 myoblasts may induce their uncontrolled proliferation and hyperplasic growth, along with inhibition of their differentiation after intramuscular transplantation [7]. To overcome this obstacle, we investigated whether paracrine properties of HO-1 overexpressing myoblasts can improve recovery from HLI in db/db mice. For this purpose we injected conditioned media from differentiated C2C12-Luc-GFP and C2C12-Luc-GFP-HO1 cells into the muscle of db/db mice 1 day after femoral artery ligation. Control animals were treated with nonconditioned medium. Measurements of blood flow revealed an improved recovery of tissue perfusion in diabetic mice treated with conditioned media harvested from the HO-1 overexpressing cells. This effect was visible at later time points, reaching statistical significance on day 28 after surgery (Figure 4A,B). Conditioned media from C2C12-Luc-GFP cells were not effective and the kinetics of blood flow restoration was similar to control mice (Figure 4A,B).

**Figure 5:**
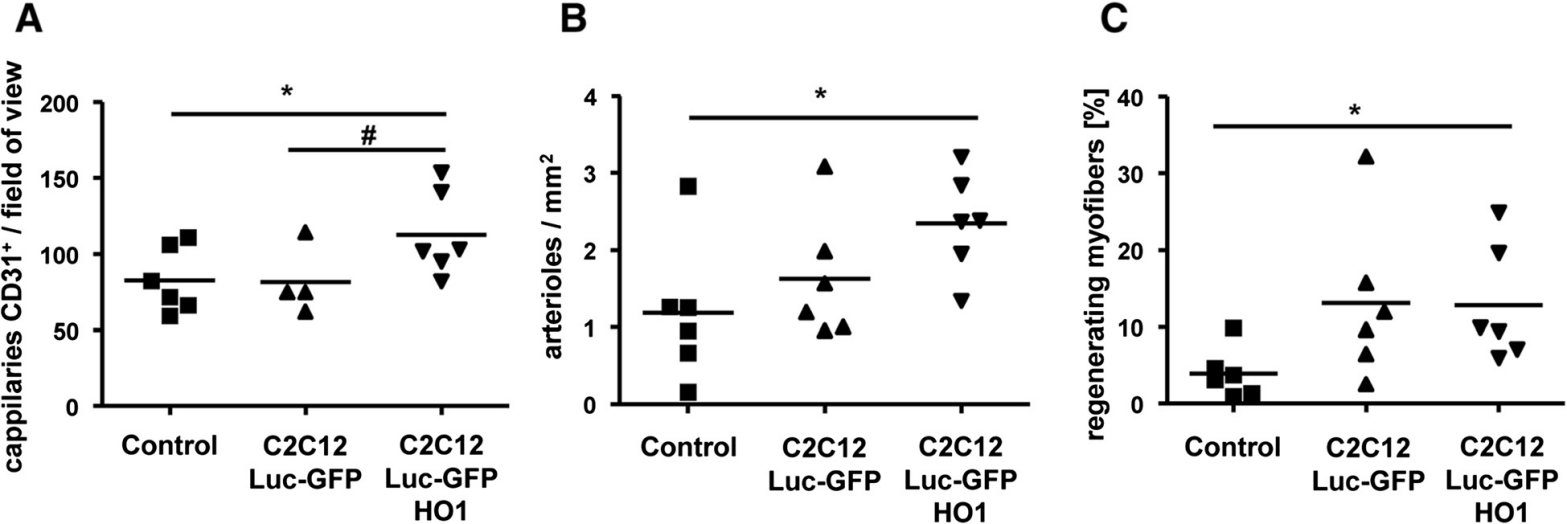
Histological and immunohistochemical analysis of ischemic muscles in hind limbs of db/db mice injected with conditioned media from C2C12-Luc-GFP, C2C12-Luc-GFP-HO1 cells or nonconditioned control media, performed on day 28 after injection of media (by quantitative analysis). **(A)** Angiogenesis – number of capillaries per microscopic field of view; immunohistochemical staining for CD31. **(B)** Arteriogenesis – number of arterioles per mm^2^; immunohistochemical staining for α-smooth muscle actin. **(C)** Myogenesis – percentage of regenerating fibers with centrally located nuclei; hematoxylin and eosin staining. Each point represents an individual animal. *P < 0.05, versus control; ^#^ *P* < 0.05, versus C2C12-Luc-GFP.

Elevated blood flow in mice treated with conditioned media from HO-1 overexpressing cells was accompanied by increased angiogenesis. The number of CD31-positive capillaries was higher in ischemic muscles of animals treated with C2C12-Luc-GFP-HO1 in comparison to those treated with control media and nonconditioned media (Figure 5A). Both arteriogenesis (assessed by analysis of αSMA-positive arterioles, Figure 5B) and myogenesis (assessed by analysis of regenerating muscle fibers with centrally located nuclei, Figure 5C) were enhanced in response to media from C2C12-Luc-GFP-HO1 cells. However, arteriogenesis and myogenesis were comparable in mice treated with conditioned media harvested from either C2C12-Luc-GFP-HO1 or C2C12-Luc-GFP cells (Figure 5B,C).

**Figure 6:**
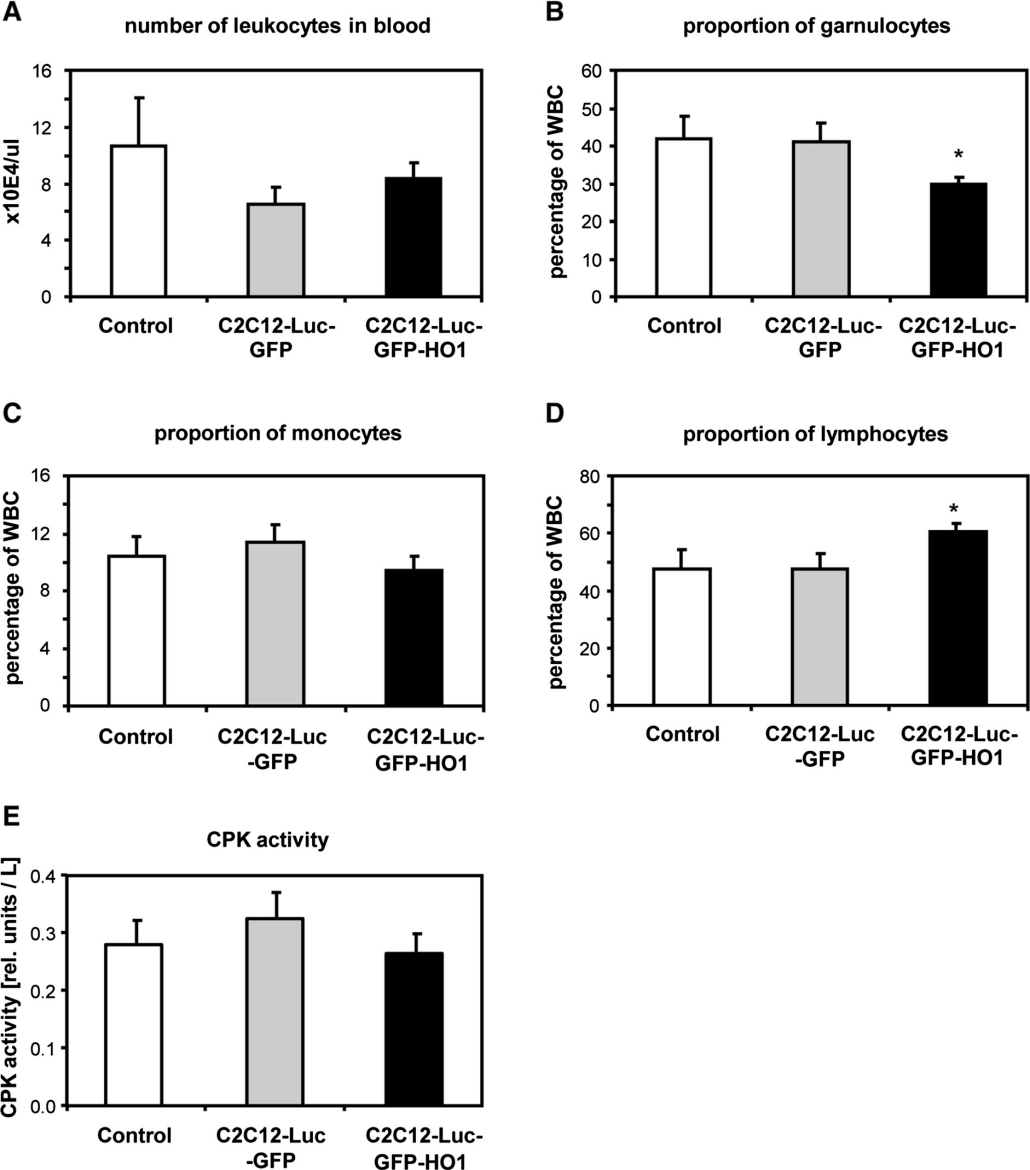
Parameters associated with inflammation and muscle degeneration analyzed on day 28 in peripheral blood of db/db mice injected with conditioned media from C2C12-Luc-GFP, C2C12-Luc-GFP-HO1 cells or non-conditioned control media. **(A)** Number of leukocytes. **(B)** Percentage of granulocytes. **(C)** Percentage of lymphocytes. **(D)** Percentage of monocytes. **(E)** Activity of creatine phosphokinase (CPK) in blood serum. Each bar represents mean + SEM (n = 5 to 6). *P < 0.05, versus control. WBC, white blood cell.

Analysis of granulocytes, monocytes and lymphocytes in peripheral blood, performed on day 28 after injection, showed that conditioned media did not influence the number of circulating leukocytes (Figure 6A). Mice treated with media harvested from the HO-1 overexpressing cells had, however, a lower proportion of granulocytes and a higher proportion of lymphocytes than those treated with control media or media harvested from C2C12-Luc-GFP cells. The proportion of monocytes was similar in all groups (Figure 6B-D). Of note, we detected did no differences in the concentration of creatine phosphokinase in the blood sera, the marker of skeletal muscle degeneration (Figure 6E).

**Figure 7:**
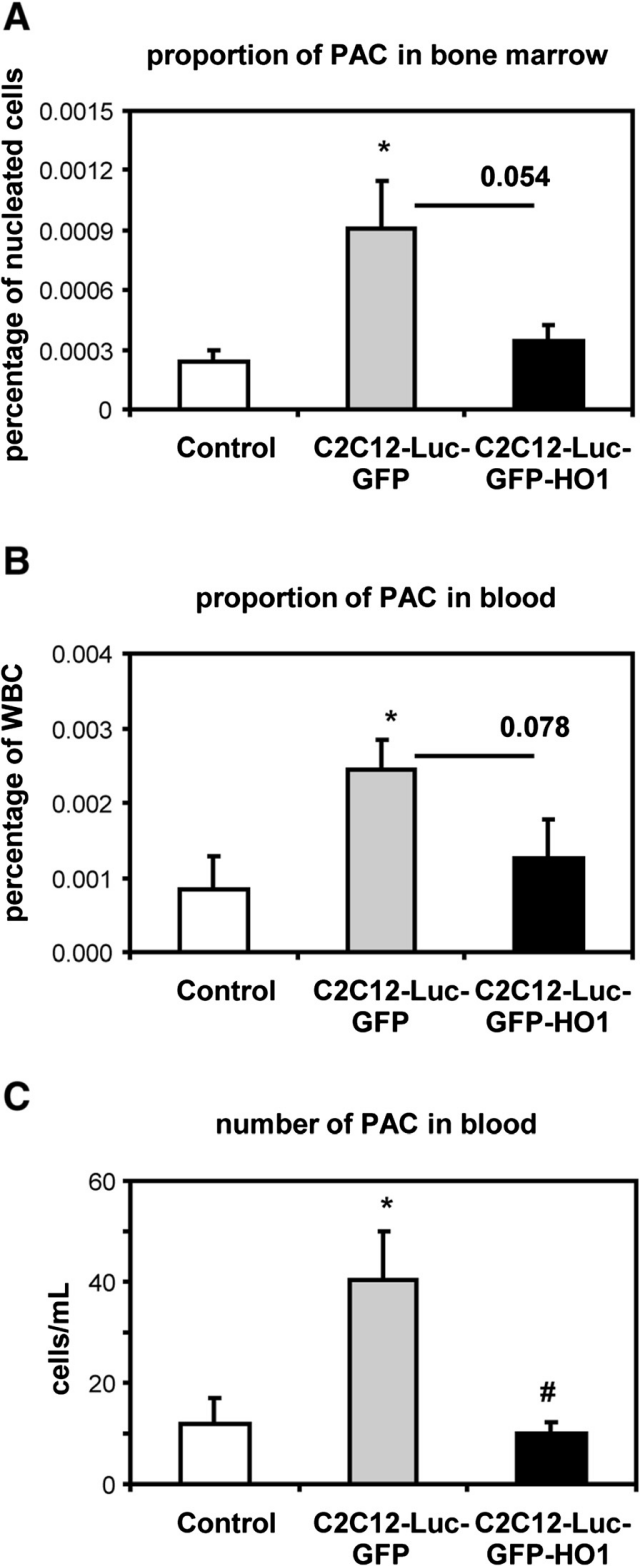
Proangiogenic progenitor cells (PACs), defined as CD45^−^KDR^+^Sca-1^+^ cells, analyzed on day 28 in peripheral blood and bone marrow of db/db mice injected with conditioned media from C2C12-Luc-GFP, C2C12-Luc-GFP-HO1 cells or nonconditioned control media. **(A)** Percentage of PACs in bone marrow. **(B)** Percentage of PACs in peripheral blood. **(C)** Number of PACs in peripheral blood. Fluorescence-activated cell sorting analysis. Each bar represents mean + SEM (n = 5 to 6). *P < 0.05, versus control, ^#^ *P* < 0.05, versus C2C12-Luc-GFP. WBC, white blood cell.

**Figure 8:**
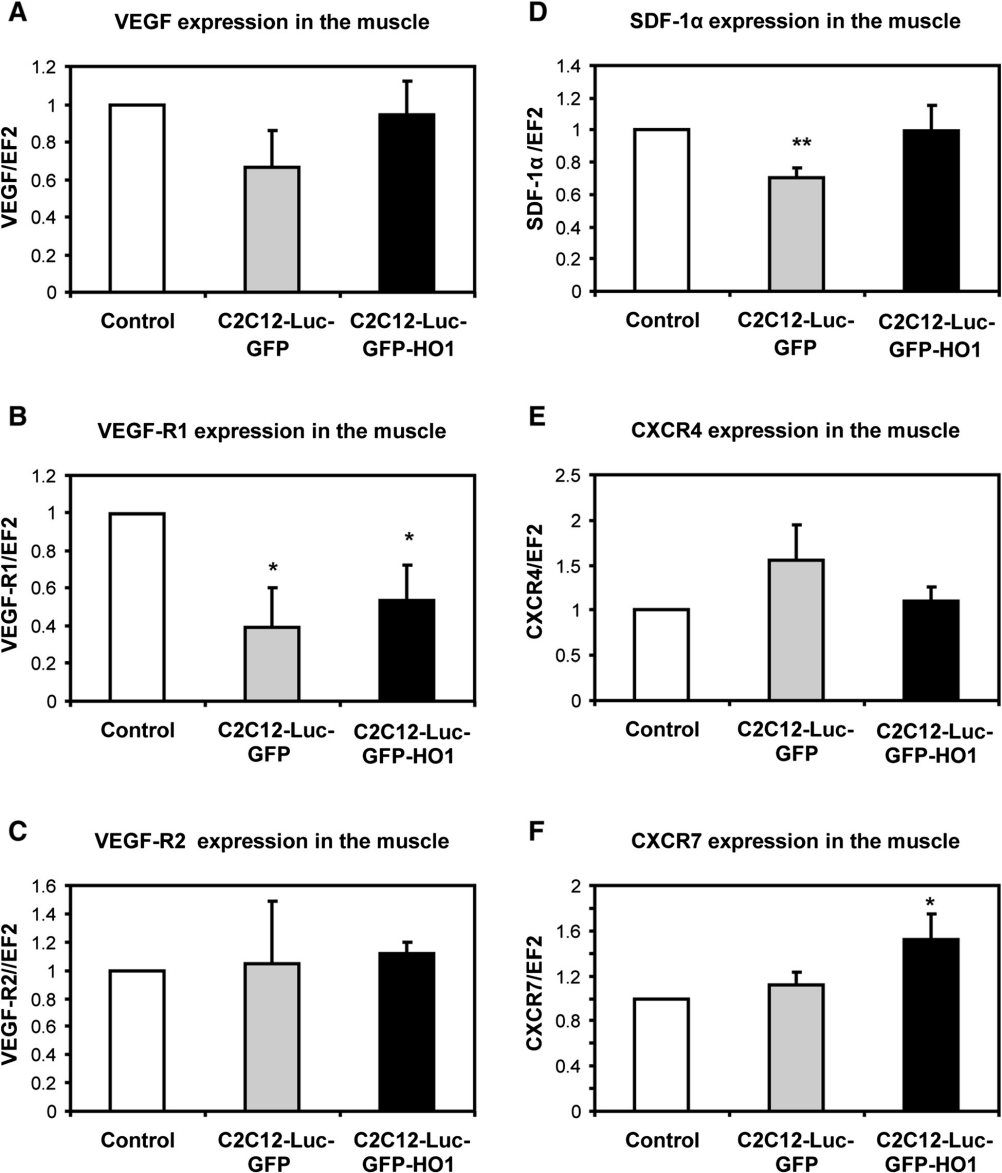
Level of VEGF, SDF-1a and their receptors in ischemic muscle. Expression of **(A)** VEGF, **(B)** VEGF-R1, **(C)** VEGF-R2, **(D)** SDF-1α, **(E)** CXCR4, and **(F)** CXCR7 mRNAs in gastrocnemius muscle of db/db mice subjected to hind limb ischemia and injected with conditioned media from C2C12-Luc-GFP, C2C12-Luc-GFP-HO1 cells or nonconditioned control media. Quantitative RT-PCR performed at day 28 after injection of media (n = 5 to 6). EF2 served as an internal control; *P < 0.05, **P < 0.01, versus control. SDF-1α, stromal cell-derived factor-1α; VEGF, vascular endothelial growth factor.

Recently we have demonstrated [23,24] that the number of circulating proangiogenic progenitor cells (PACs; defined as CD45^−^KDR^+^Sca-1^+^ [23] or KDR^+^CXCR4^+^ [24]) does not correlate with restoration of blood flow in ischemic muscles. In accordance, in the current study we also did not observe such a connection. On day 28 of the experiment, the perfusion of muscle was highest in mice treated with media from HO-1 overexpressing cells, while similarly lower in both animals treated with unconditioned media or that injected with conditioned media from control cells (Figure 4). Fluorescence-activated cell sorting analysis performed on day 28 in the same individuals showed that the proportion of CD45^−^KDR^+^Sca-1^+^ PACs in the bone marrow, as well as their proportion and number in peripheral blood, was higher in mice treated with conditioned media from control cells than in two other groups (Figure 7). Similarly, gene expression of VEGF and SDF-1α pathways in the total skeletal muscle did not show any clear connection with blood flow restoration (Figure 8). Expression of VEGF, VEGF-R2, and CXCR4 were similar in all groups. Both groups treated with conditioned media had lower level of VEGF-R1, while only mice treated with conditioned media from control cells also showed reduced expression of SDF-1α. Mice injected with media from HO-1 overexpressing cells showed higher levels of CXCR7.

## Discussion

Functional impairment of skeletal muscles in diabetes includes not only insulin resistance, ischemia, lipid accumulation, reduced mass, force and oxidative capacity [25], but also disturbed regeneration after injury [11,26]. The latter can be attributed, at least in part, to impaired function of mSCs, which are less abundant in diabetic conditions [27] and show reduced activation as well as weakened differentiation into mature myotubes [16]. Diabetic mSCs can induce pathological adipogenesis instead of myogenesis [17] and are more prone to oxidative stress [18]. Molecular characteristics of differences between diabetic mSCs and their healthy counterparts in the context of myogenic markers has not yet been examined.

To the best of our knowledge, we have revealed for the first time that mSCs of db/db mice express less Pax7 and MEF2 - transcription factors associated with quiescent mSCs [8,9]. On the contrary, Myf5 and CXCR4 characteristic for activated mSCs [8,9] were elevated in cell cultures of primary mSCs isolated from diabetic individuals. Interestingly, mSCs of db/db mice have decreased expression of the major differentiation markers myoD and myogenin [8,9], which proves their impaired differentiation potential. This, together with a higher growth rate of diabetic mSCs observed in our cell cultures, could be attributed to elevated expression of CXCR4, the major receptor for SDF-1α which is a potent mitogen for mSCs [28,29].

It was shown previously that during *in vivo* regeneration of diabetic muscle, 5 days after injury the myogenin expression in skeletal muscle tissue was inhibited [26], as well the number of myoD^+^ cells being diminished [11]. This may be a consequence of disturbed function of diabetic mSCs revealed in our experiments.

It is also worth mentioning that disturbance in regeneration of diabetic muscle after injury is strictly accompanied by impaired revascularization and lower capillary density in db/db mice [11]. This correlation is not surprising, since myogenesis and angiogenesis during muscle regeneration influence each other. Formation of new blood vessels and muscle fibers occur simultaneously [30], and regeneration of a destroyed muscle involves tissue revascularization [22]. Moreover, more than 80% of muscle progenitor cells are not further than 20 μm from capillaries, and the higher the vascularization of the muscle is, the more mSCs are present [31]. Such a high co-localisation was not even evident between mSCs and nuclei of mature muscle cells [31].

Angiogenesis may be induced by mSCs and myoblasts, which secrete proangiogenic growth factors [31,32]. Proangiogenic properties are also characteristic for HO-1, since its activation may stimulate production and activity of VEGF and SDF-1α [1-3,33,34]. Injection of HO-1 overexpressing mesenchymal stem cells into ischemic heart improved neovascularization [35]. Recently we have shown similar effects after injection of plasmid encoding hypoxia-regulated HO-1 into hind limb muscle after a femoral artery ligation procedure [6]. Importantly, the proper level of HO-1 seems to be important for blood flow restoration in wounded skin or ischemic muscles of diabetic mice [4,24].

Acellular therapies, exploiting conditioned media from HO-1 overexpressing cells, have so far been shown to induce angiogenic activities of endothelial cells [2], protect kidneys from damage after cisplatine treatment [36], or induce neovascularization in ischemic heart [34]. Conditioned media were also beneficial in the treatment of diabetic wounds [37] and regeneration of blood flow after ischemia in diabetic mice [24] more effectively than were the cells. However, whether it is important to provide high expression of HO-1 in cells producing the conditioning media that is subsequently used for treatment of ischemic diabetic muscle, and whether the therapeutic effect is stable, remain so far unexamined. Answering these questions was the aim of our study.

Conditioned media used for experiments were prepared from myoblasts of the C2C12 cell line, modified to overexpress control genes (Luc and GFP) or additionally HO-1 [7]. A cell line-based model was chosen since production of cytokines is thought to be more reproducible between cell cultures than between cultures of primary mSCs. Conditioned media used for experiments were collected from myoblasts differentiating C2C12 cells, since the maturing mSCs display proangiogenic properties [31]. Accordingly, we have observed elevated levels of SDF-1α during differentiation. The media were injected into gastrocnemius muscle of db/db mice, following double femoral artery and vein ligation and induction of ischemia.

Media harvested from C2C12-Luc-GFP-HO1 cells promoted angiogenesis and improved blood flow in ischemic muscle to a higher extent than did the control media. In animals treated in this manner the blood flow was continuously improving throughout the time course of the experiment and, on day 28, was at the same level as in the intact leg. In accordance, lack of HO-1 hampers blood flow regeneration in ischemic heart of diabetic mice [38], whereas its induction improves neovascularization in the heart of healthy animals [33]. In mice treated with unconditioned media or media from C2C12-Luc-GFP cells, blood flow was recovering during the first 2 weeks of the experiment; at that point the perfusion of the ischemic leg was stable and reached only 50% to 60% of values typical in intact muscle. Interestingly, a similar disturbance in neoangiogenesis with decreased capillary density leading to reduced blood flow 14 days after HLI surgery was described previously in diabetic mice [15,39,40]. Healthy individuals were able to improve blood flow throughout a 4-week experiment, reaching values of perfusion close to normal on day 28 [15,39,40]. On this basis, we suppose that the paracrine effect of HO-1 overexpression in C2C12 myoblasts improves blood vessel regeneration in db/db mice to the same extent as in wild-type, normoglycemic mice [15,39,40]. Disturbances in blood flow regeneration of diabetic animals were observed in comparison to heterozygotic db^+^/db^−^ mice as well, although with slightly different kinetics of blood flow recovery and complete restoration after 4 weeks [41,42]. The reasons for this could be technical differences, since HLI can be performed only by femoral artery ligation [41,42], femoral artery ligation and excision of additional artery branches [15,39,40], and femoral and vein ligation (as in our experiments), which can possibly affect the level of blood flow restoration.

Treatment of ischemic muscle with conditioned media from C2C12-Luc-GFP-HO1 cells significantly induced regeneration of muscle tissue in comparison to animals injected with unconditioned media, as indicated by the increased number of regenerating myofibers. Interestingly, similar enhancement, which did not reach statistical significance, was also observed in the case of animals treated with media conditioned by C2C12-Luc-GFP cells. It can be hypothesized that such an effect of myoblast-conditioned media can be attributed to growth factors, which are known to be secreted by these cells (for instance, from fibroblast growth factor [43] or insulin-like growth factor [44] families). They are able to induce proliferation and/or differentiation of myoblasts in a paracrine manner [43,44] and therefore can also enhance regeneration.

It is worth mentioning that the proposed therapeutic approach did not induce inflammatory reaction. Interestingly, we observed a decreased percentage of granulocytes in blood after treatment of ischemic muscle with C2C12-Luc-GFP-HO1 conditioned media. This seems to be in accordance with the anti-angiogenic role of neutrophils during wound healing in db/db mice [45,46], evidenced for example by the inverse correlation between neutrophil accumulation in injured tissue and the number of new blood vessels [47].

Moreover, in contrast to implantation of modified myoblasts engineered to overexpress HO-1, which may lead to uncontrolled proliferation without differentiation [7], the conditioned media strategy did not result in formation of hyperplasic tissue. Thus, this method seems to be safer than a cell-transplantation approach.

Muscle progenitor cells secrete VEGF [31,32,48], which is a main growth factor responsible for mSC-induced angiogenesis both *in vitro* [32] and *in vivo* [49], and also after ischemia [48]. VEGF is a key player regulating development of a proper microcirculation system in skeletal muscle [50]. Its expression was reported to be lowered in diabetic animals leading to disturbed neovascularization [14]. However, VEGF is not responsible for observed *in vivo* induction of angiogenesis in our experimental setting, since its expression was unchanged between muscles injected with C2C12-Luc-GFP and C2C12-Luc-GFP-HO1 conditioned media. Additionally, expression of VEGF in C2C12 cells overexpressing HO-1 cultured under differentiating conditions was even decreased.

On the other hand, we have previously shown that myoblasts overexpressing HO-1 secrete more SDF-1α [7]. In this study we have confirmed that expression and secretion of SDF-1α is increased in C2C12-Luc-GFP-HO1 cells, especially during differentiation. This cytokine is a well known proangiogenic factor, and HO-1 is necessary for its proper action [3,33,36]. SDF-1α stimulates proliferation and migration of mature endothelial cells, inducing them to form tube-like structures [3]. The mechanisms of these actions involves phosphorylation of cytoskeletal vasodilator-stimulated phosphoprotein, for which the proper level of CO obtained due to HO-1 activity is critical [3]. Additionally, SDF-1α is known to stimulate expression of E-selectin, inducing adhesion of endothelial cells and formation of capillaries [51]. Dysfunction of SDF-1α secretion may lead to hampered neovascularization of ischemic muscle in diabetic mice [21].

We suppose that SDF-1α could be a main factor in C2C12-Luc-GFP-HO1 conditioned media improving the induction of neovascularization in ischemic muscle of db/db mice. A similar effect was obtained in ischemic heart, where transplantation of myoblasts overexpresssing SDF-1α upregulated angiogenesis and improved functional parameters [52]. Cell therapy with fibroblasts overexpressing SDF-1α also facilitated wound healing by induction of neovascularization [51].

Apart from regulating angiogenesis at the level of mature endothelial cells, HO-1 and SDF-1α also increase migration of proangiogenic progenitor cells into a wound site, and decrease their apoptosis while increasing proliferation and migration, thereby accelerating regeneration of the microcirculation [3,20,24]. Exogenous SDF-1α injected intramuscularly [53], delivered by a gene transfer to mature muscle cells [54], or introduced *ex vivo* into proangiogenic progenitors before transplantation [55] also improved neovascularization in ischemic skeletal muscles. Importantly, decreased mobilization of PACs observed in diabetes [14,15] can be caused by decreased HO-1/SDF-1α expression [12,21] or by impaired response to SDF-1α [12,13]. This may result in impaired neovascularization during wound healing [12] and regeneration of blood flow in ischemic skeletal muscle [13,21]. It was also shown that exogenous SDF-1α improved mobilization and migration of PACs, leading to accelerated healing of diabetic wounds [12], whereas activation of the HO-1/SDF-1α pathway enhanced PAC mobilization and promoted re-endothelialization of the carotid artery in diabetic mice [56].

Our experiment does not allow us to evaluate the effect of conditioned media on mobilization of PACs at early time points after ischemia. Analysis performed at day 28 did not show, however, any association between PACs in bone marrow or peripheral blood and restoration of blood flow. It cannot be excluded that such a relationship could be present soon after induction of ischemia, where mobilization of proangiogenic progenitors from the bone marrow is most pronounced [12,15,21,51]. Nevertheless, in our earlier study performed in hyperglycemic mice with normal or reduced expression of HO-1, we did not observe any connection between circulating PACs and tissue reperfusion at early time points [24].

SDF-1α is not the only possible mediator of paracrine effects of HO-1 overexpressing C2C12 cells on blood flow recovery in ischemic muscles. Mass spectrometry analysis of the composition of conditioned media harvested from C2C12-Luc-GFP and C2C12-Luc-GFP-HO1 cells revealed other secreted proteins which might facilitate neovascularization. In media from HO-1 overexpressing cells, concentrations of proagniogenic factors such as peptidyl-prolyl cis-trans isomerase A, macrophage migration inhibitory factor, haptoglobin and galectin-1 were increased, accompanied by decreased levels of the angiogenesis inhibitor pigment epithelium-derived factor. Since they affect neovascularization [57-61], these factors may also potentially facilitate tumor development [58,62-64]. Importantly, paracrine effects of HO-1 overexpression in C2C12 cells did not lead to uncontrolled hyperplasic growth of myoblasts in ischemic muscle, in spite of increased proliferation and disturbed differentiation observed earlier in HO-1 overexpressing C2C12 cells [7]. Instead, we observed a tendency toward increased number of regenerating fibers in muscles treated with media harvested from C2C12-luc-GFP and C2C12-luc-GFP-HO1 cells. One could speculate on the role of promyogenic proteins such as biglycan, fibronectin, follistatin-related protein-1 or galactin-1 [65-67], which were elevated in conditioned media.

## Conclusions

In conclusion, we have shown that overexpression of HO-1 in myoblasts may augment paracrine proangiogenic potential of these cells. Therapy with conditioned media can lead to accelerated angiogenesis in ischemic muscles of db/db mice, without inducing inflammatory reaction and uncontrolled proliferation of myoblasts.

## Abbreviations

αSMA: α-smooth muscle actin
CO: carbon monoxide
DM: differentiation medium
FCS: fetal calf serum
GM: growth medium
HLI: hind limb ischemia
HO-1: heme oxygenase-1
mSC: muscle satellite cell
myoD: myogenic differentiation antigen
PAC: proangiogenic progenitor cell
SDF-1α: stromal cell-derived factor-1α
VEGF: vascular endothelial growth factor
